# IMPACT OF OMA ON HEALTH CARE TRAINEE ATTITUDES AND PERCEPTIONS TOWARD PEOPLE LIVING WITH DEMENTIA

**DOI:** 10.1093/geroni/igae098.1507

**Published:** 2024-12-31

**Authors:** Diane Berish, Jennifer Rank, Janice Whitaker, Daniel George

**Affiliations:** The Pennsylvania State University, University Park, Pennsylvania, United States; The Pennsylvania State University, University Park, Pennsylvania, United States; The Pennsylvania State University, University Park, Pennsylvania, United States; The Pennsylvania State University, University Park, Pennsylvania, United States

## Abstract

To meet the complex needs of the growing population of people living with dementia (PLwD), the healthcare workforce must have not only the necessary clinical knowledge, but also the interpersonal skills. Experiences in the arts and humanities, such as OMA, can provide opportunities to acquire best-practice approaches. At present, no evaluation of OMA has been conducted on health trainees. We implemented OMA at Penn State University in both the Nese College of Nursing (NCON) and the College of Medicine (COM). In the NCON, OMA was implemented into nursing clinicals and as part of a memory cafe. N=10 clinical and N=15 memory café students completed pre and post assessments (Allophilia, Dementia Attitudes) of their perceptions and attitudes towards PLwD. Results showed that clinical students reported higher levels of comfort with PLWD on both scales (Allophilia, p=.0039; Dementia Attitudes, p=.002). Memory café students reported higher Allophilia scores (affection, p=.02; comfort, p=.04; engagement, p=.02) and Dementia Attitudes knowledge (p=.02). The COM was one of 6 medical schools to implement OMA in 2019-2020. Overall, 52 first- or second-year medical students were trained in OMA and participated with residents at partner assisted living facilities. Students’ scores showed significantly larger pre-post improvement than controls with respect to Dementia Attitudes scores (p=.001) and all Allophilia-scale factors (p=.001). Our work suggests that arts-based learning experiences such as OMA, which enable playful, creative, humanizing interactions between health trainees and PLwD, may foster more positive attitudes. Experiences like OMA might improve attitudes and encourage students to pursue geriatric specialties.

